# Screening and brief alcohol intervention in routine primary care in the UK: 12-month outcomes

**DOI:** 10.1186/1940-0640-7-S1-A81

**Published:** 2012-10-09

**Authors:** Eileen Kaner, Colin Drummond, Paolo Deluca, Dorothy Newbury-Birch, Simon Coulton

**Affiliations:** 1 Institute of Health and Society, Newcastle University, Newcastle upon Tyne, UK; 2 National Addiction Center, Institute of Psychiatry, King's College London, London, UK; 3 Institute of Psychiatry, King's College London, London, UK; 4 Center for Health Service Studies, University of Kent, Canterbury, UK

## 

Numerous brief intervention (BI) trials have reported positive effects in primary care. However, it is unclear if structured advice or counseling is the more effective form of BI. The Screening and Intervention Program for Sensible Drinking (SIPS) trial aimed to evaluate the cost-effectiveness of different intensities of BI at reducing risky drinking in primary care. Practices were randomly allocated to one of three conditions: a leaflet-only control; five minutes of brief structured advice; or 20 minutes of brief counseling. Practices were asked to recruit at least 31 risk drinkers who received a short assessment followed by BI. Patients were followed up at six and 12 months post-intervention. The primary outcome was the proportion of risky drinkers as measured by the Alcohol Use Disorders Identification Test (AUDIT). Overall, 3562 patients were assessed for eligibility in 29 practices: 2991 (84%) were eligible; 900 (30%) screened positive for risky drinking; and 752 (83.6%) consented to participate in the trial. At 12 months, 79% patients (n = 598) were available for follow-up. No significant differences in follow-up rates were observed by condition. There was an overall reduction in risky drinking of 16.5% between baseline and 12 months. By condition, the reductions were 17.3% for controls, 12.7% for brief advice, and 19.6% for brief counseling. An adjusted logistic regression model identified baseline AUDIT score and gender as significant predictors of risky drinking at 12 months. Patients with lower baseline scores and women were more likely to be negative for risky drinking at follow-up based on AUDIT score. Brief advice and brief counseling did not produce significantly greater effects in reducing risky drinking than leaflet-only. We discuss these findings in light of the current BI literature.

